# Elektrokonvulsionstherapie bei Menschen mit Intelligenzminderung

**DOI:** 10.1007/s00115-024-01713-6

**Published:** 2024-08-21

**Authors:** M. Guhra, S. H. Kreisel, D. Zilles-Wegner, A. Sartorius, T. Sappok, N. Freundlieb

**Affiliations:** 1 Medizinische Fakultät und Universitätsklinikum OWL, Evangelisches Klinikum Bethel, Universitätsklinik für Psychiatrie und Psychotherapie, Abt. für Gerontopsychiatrie, Universität Bielefeld, Bielefeld, Deutschland; 2 Klinik für Psychiatrie und Psychotherapie, Universitätsmedizin Göttingen, Göttingen, Deutschland; 3 Klinik für Psychiatrie und Psychotherapie, Medizinische Fakultät Mannheim/Universität Heidelberg, Zentralinstitut für Seelische Gesundheit, Mannheim, Deutschland; 4 Medizinische Fakultät und Universitätsklinikum OWL, Krankenhaus Mara, Universitätsklinik für Inklusive Medizin, Universität Bielefeld, Bielefeld, Deutschland; 5 MZEB Berlin-Nord der GIB-Stiftung, Berlin, Deutschland Germanenstr. 33, 13156

**Keywords:** Elektrokonvulsionstherapie, Intelligenzminderung, Psychische Störung, Autismusspektrumstörungen, Katatonie, Electroconvulsive therapy, Intellectual disability, Mental disorder, Autism spectrum disorder, Catatonia

## Abstract

Die Elektrokonvulsionstherapie (EKT) ist eine hochwirksame Behandlungsoption bei schweren psychischen Erkrankungen. Für Menschen mit Intelligenzminderung (IM) sind die Zugangswege zur EKT bis heute erschwert, obwohl sie mindestens so häufig wie die Normalbevölkerung an psychischen Erkrankungen leiden. Im Rahmen einer systematischen Literaturrecherche und anhand eines Fallberichtes wird die wissenschaftliche Grundlage für eine EKT-Behandlung von Menschen mit IM dargestellt. Insgesamt wurden 100 Artikel mit 208 Fallberichten recherchiert und ausgewertet. Zusammengefasst sprechen diese für eine mindestens gleich gute Wirkung und ähnliche Nebenwirkungsrate im Vergleich zur Behandlung von Personen ohne IM. Neben schweren affektiven und psychotischen Störungen werden vor allem katatone Syndrome bei Menschen mit IM erfolgreich mit EKT behandelt. Eine EKT kann dabei die Lebensqualität der Patient:innen bessern und stellt nicht selten eine lebensrettende Behandlung dar. Die Rückfallprophylaxe sollte möglichst früh im Therapieprozess eingeplant werden. Leichtere Zugangsbedingungen zu einer EKT-Behandlung für Menschen mit IM zu schaffen, entspricht der wissenschaftlichen Datenlage und auch dem Recht auf eine gleichwertige Behandlung gemäß der UN-Behindertenrechtskonvention Artikel 25.

**Zusatzmaterial online:**

Zusätzliche Informationen sind in der Online-Version dieses Artikels (10.1007/s00115-024-01713-6) enthalten.

## Abstract (en)

Electroconvulsive therapy (ECT) is a highly effective treatment option for severe mental illness. Although people with intellectual disability (ID) have similar prevalence rates of mental disorders in comparison to the general population their access to ECT remains challenging. A systematic literature review was carried out on treatment with ECT in patients with ID and a case report on a patient with ID who underwent ECT is presented, to highlight a typical clinical routine. A total of 100 articles with 208 different case reports were retrieved. In summary, the results underline the effectiveness of ECT in people with ID, with side effects comparable to those in the general population. The ECT is effective in the treatment of severe affective and psychotic disorders and particularly in people with catatonia. The use of ECT can improve the patient’s mental health and quality of life and is often a life-saving treatment option. The prophylaxis of relapses should be included as early as possible in the planning process. Providing an easy access to ECT treatment for people with ID is corroborated by its effectiveness and is in line with the right to equal treatment in accordance with article 25 of the United Nations Convention on the Rights of Persons with Disabilities.

## Hintergrund

Die Elektrokonvulsionstherapie (EKT) findet vor allem Einsatz bei affektiven und psychotischen Störungen mit einer Ansprechrate von 50–80 % [1]. Die Prävalenz schwerer psychischer Erkrankungen bei Menschen mit Intelligenzminderung (IM; in der ICD-11 „Störung der Intelligenzentwicklung“) ist mindestens so hoch wie in der Normalbevölkerung [2, 3]. Dennoch wird die EKT bei diesem Personenkreis nur selten angewandt. Die Gründe dafür sind vielfältig. Zum einen kann die Diagnosestellung psychischer Störungen, bei denen eine EKT indiziert sein kann, bei Menschen mit IM erschwert sein. Darüber hinaus sind Planung und Durchführung einer EKT bei nicht oder nur eingeschränkt einwilligungsfähigen Patient:innen mit oftmals komplexen Verhaltensauffälligkeiten mit einem hohen Aufwand verbunden. Das Verhalten und der besonders hohe Bedarf an individueller Betreuung sprengt häufig die Grenzen der organisatorischen Möglichkeiten von Krankenhäusern, die eine EKT anbieten. Je nach lokaler Rechtslage kann der Zugang auch aus juristischen Gründen erschwert sein [4].

In diesem Artikel wird auf dem Boden eines selektiven Literaturreviews die vorhandene Datenlage zusammengefasst. Folgende Fragen sollen beantwortet werden: Welches sind die Hauptindikationen für eine EKT bei Menschen mit IM, wie ist die Wirksamkeit und welche Nebenwirkungen werden berichtet?

## Methodik

Wir führten zuletzt am 20.06.2023 eine Literaturrecherche in PubMed mit den Suchbegriffen (ECT OR electroconvulsive therapy) AND (intellectual OR autism OR retardation OR mental OR developmental OR disability) durch.

Einschlusskriterien waren (1) Peer-reviewed-Artikel über EKT bei Menschen mit IM und (2) Artikel, die in Englisch, Deutsch oder Niederländisch publiziert wurden. Im englischsprachigen Kontext ist der Begriff „learning disability“ (Lernbehinderung) weitverbreitet und wird teils alternativ zu „intellectual disability“ (Intelligenzminderung) genutzt. Vor diesem Hintergrund wurden auch Artikel über EKT bei Menschen mit einer Lernbehinderung eingeschlossen, auch wenn strenggenommen eine Intelligenzminderung erst unterhalb eines Intelligenzquotienten (IQ) von 70 diagnostiziert wird.

**Abb. 1:**
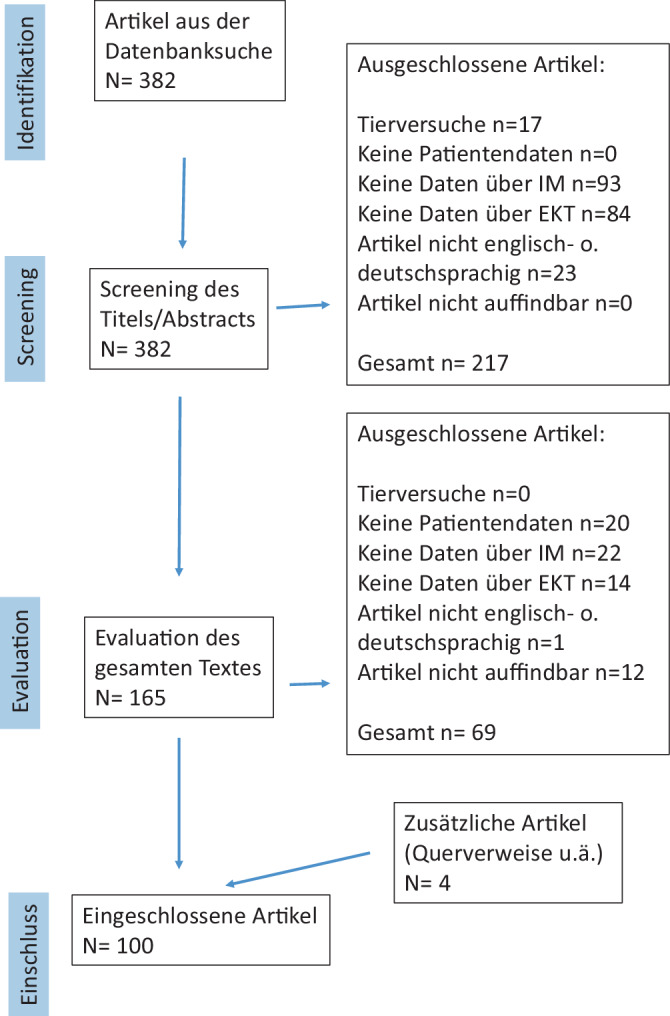
Flussdiagramm der Literaturrecherche. *EKT* Elektrokonvulsionstherapie, *IM* Intelligenzminderung; *N* Anzahl

Die Referenzliste der identifizierten Artikel wurde auf weitere relevante Literatur durchsucht. Von den 382 identifizierten Artikeln wurden 286 ausgeschlossen, weil sie Tierversuche, keine Patientendaten, keine EKT oder keine Menschen mit IM behandelten oder nicht auffindbar waren (Abb. 1). Diese Studie umfasst die Ergebnisse von 100 Artikeln (Kasuistiken und Fallserien) mit insgesamt 208 Fallbeschreibungen.

Die Ergebnisse werden als qualitative Zusammenfassungen der Evidenz für die häufigsten EKT-Indikationen entsprechend der aktuellen Stellungnahme der DGPPN [5] zusammengefasst.

Zusätzlich wird ein Fallbericht vorgestellt, um praxisnah einen typischen Behandlungsverlauf zu veranschaulichen.

## Ergebnisse

Randomisierte kontrollierte Studien für den Einsatz der EKT bei Menschen mit IM liegen nicht vor. Es fanden sich lediglich Kasuistiken und Fallserien, aus denen die Charakteristika der behandelten Patient:innen ermittelt wurden (s. Supplement-eTabelle 1). Tab. 1 gibt einen Überblick über die demografischen Daten der eingeschlossenen Personen. Das Alter der Behandelten lag im Mittel bei 27,7 Jahren, bei 32 Fällen fehlten die Altersangaben. Es wurden mehr männliche als weibliche Patient:innen behandelt (58,1 vs. 41,9 %), wobei erstere im Mittel jünger waren (24,9 vs. 31,7 Jahre). In 75 Fällen fehlten die Angabe zum Schweregrad der IM, die übrigen 133 Patient:innen hatten in über 80 % der Fälle eine leichte bis mittelgradige IM (Tab. 2). Angaben zur Ätiologie der IM lagen in der Mehrzahl der Fälle nicht vor. In 81 Fällen (38,9 %) wurde eine Ursache der IM bzw. ein(e) mit der IM assoziierte(s) Erkrankung/Syndrom angegeben. In der überwiegenden Zahl war dies mit 50 Fällen (61,7 %) eine Autismusspektrumstörung (ASS), gefolgt vom Down-Syndrom mit 17 Fällen (21 %). Andere spezifizierte Diagnosen machen mit 14 Fällen 17,3 % aus (s. Supplement-eTabelle 1).

**Tab. 1:** Demografische Daten

		***N***	**%**			
*Geschlecht*	Weiblich	85	41,9			
	Männlich	118	58,1			
	Angabe fehlt	5				
*–*		***N***	**Mittelwert**	**SD**	**Min**	**Max**
*Alter*		176	27,7	14,7	8	71
	Angabe fehlt	32				
*Alter vs. Geschlecht*	Weiblich	71	31,7	15,3	12	69
	Männlich	105	24,9	13,6	8	71
	Angabe fehlt	27				

*Max* Maximum, *Min* Minimum, *N* Anzahl, *SD* Standardabweichung

**Tab. 2:** Grad der Intelligenzminderung

	*N*	%
Lernbehinderung („borderline“)	8	6,0
Lernbehinderung-leicht („borderline-mild“)	1	0,8
Leicht („mild“)	63	47,4
Leicht-mittelgradig („mild-moderate“)	4	3,0
Mittelgradig („moderate“)	40	30,1
Mittelgradig-schwer („moderate-severe“)	1	0,8
Schwer („severe“)	6	4,5
Schwerst („profound“)	10	7,5
Spezifische Angabe fehlt	75	–

In Klammern stehen die entsprechenden Begrifflichkeiten in den überwiegend englischsprachigen Publikationen

*N* Anzahl

In 189 Fällen (90,9 %) wurde eine Besserung der Symptomatik berichtet, in 17 Fällen änderte sich die Symptomatik nicht, und bei zwei Patient:innen wurde eine Verschlechterung der Symptomatik angegeben. Letztere wurden unter den Indikationen Psychose [6] bzw. bipolare Störung und malignes neuroleptisches Syndrom (MNS; [7]) behandelt, die Fallberichte stammen aus den 1980er-Jahren. Unter den Fällen ohne Veränderung der Symptomatik fanden sich neben 2 Patient:innen mit der Diagnose „intermittent explosive disorder“ [8] lediglich Fälle mit den Diagnosen Psychose/Schizophrenie [6, 9–13], schizoaffektive Störung [14], Katatonie [9, 12–16] oder MNS [14], teilweise in Kombination, aber keine mit der Indikation einer affektiven Störung oder einer Verhaltensstörung.

Die Anzahl an Einzelbehandlungen in einer Serie wird zwischen 7 und 29 angegeben [17–19] mit einer Frequenz von 2 bis 3 Behandlungen pro Woche. Eine daran anschließende Erhaltungs-EKT wird zwischen wöchentlich bis zu einmal alle 2 bis 3 Wochen entsprechend dem individuellen Befund für eine theoretisch unbegrenzte Zeitspanne durchgeführt [20, 21]. Viele Fallberichte schildern ein rasches Wiederauftreten der Symptomatik schon nach temporärer Unterbrechung [20, 22–24]. Als häufigste Elektrodenposition wird von einer bilateralen/bitemporalen Stimulation berichtet.

Die Indikationen zur EKT (Tab. 3) entsprachen im Wesentlichen denen bei Menschen ohne IM, jedoch in anderer Wichtung: Die häufigste Indikation war die Katatonie (37,1 %). An zweiter Stelle finden sich mit 29,3 % die affektiven Störungen, wobei unter diesen die unipolare depressive Episode die häufigste Diagnose darstellt, während bei der Indikation „bipolare Störung“ häufig nicht differenziert wurde, ob es sich um eine depressive, manische oder gemischte Episode handelte. In einer Publikation ist lediglich von „affektiven Störungen“ die Rede [8]. Es folgen die Störungen aus dem psychotischen Spektrum mit den Diagnosen „Psychose“ oder „Schizophrenie“ und der schizoaffektiven Störung – auch hier wurde in der Regel nicht zwischen den einzelnen Episoden unterschieden. Eine Indikation, die sich bei Menschen ohne IM mit Ausnahme von Einzelfallberichten bei Patient:innen mit Demenz [5] nicht findet, sind therapieresistente schwere Verhaltensstörungen, wobei es sich dabei im Wesentlichen um schweres selbstverletzendes Verhalten (SVV) handelt, die 10,6 % der Indikationen darstellen. Seltene Indikationen (kumulativ 5,1 %) waren die Manie ([25, 26]; ohne Angabe einer bipolaren Störung), das MNS [7, 14, 27–29] und in einzelnen Fällen ein Delir [30] und medikationsrefraktäre epileptische Anfälle [31]. In einigen Fällen wurden für einzelne Patient:innen mehrere Indikationen angegeben.

**Tab. 3:** EKT-Indikationen

			Alter^a^		Anzahl Publikationen
	*N*^b^	%	Mittelwert	SD	
Katatonie	95	37,1	22,0	9,4	56
Depression	34	13,3	36,3	15,5	25
Bipolare Störung	29	11,3	31,3	17,0	23
Affektive Störungen^c^	12	4,7	–	–	1
Psychose/Schizophrenie	36	14,1	29,2	15,1	19
Schizoaffektive Störung	10	3,9	37,8	15,8	10
Verhaltensstörungen	27	10,6	13,2	5,0	16
Sonstige spezifizierte Indikationen^d^	13	5,1	34,6	19,6	11

*EKT* Elektrokonvulsionstherapie, *N* Anzahl, *SD* Standardabweichung

^a^ Werte beziehen sich nur auf Probanden, bei denen spezifische Angaben vorhanden waren

^b^ Aufgrund von Mehrfachangaben ergibt sich eine Gesamtanzahl von 256 Indikationen

^c^ Die Indikation „affektive Störung“ wurde in einer Studie [8] angegeben, ohne dass weiter differenziert wurde

^d^ Zu den sonstigen spezifizierten Indikationen s. die Supplement-eTabelle 1

Im Folgenden gehen wir auf die wichtigsten Indikationen gesondert ein.

### Affektive Störungen

In internationalen Metaanalysen wird berichtet, dass Patient:innen mit IM mit 6,7 % mindestens gleich häufig an affektiven Störungen leiden wie die durchschnittliche Bevölkerung [32–34], in einer Auswertung deutscher ambulanter Behandlungsdaten wurde sogar bei fast jeder 4. Person mit IM eine affektive Störung diagnostiziert [35]. Eine Besserung schwerer, therapieresistenter Depressionen durch EKT unabhängig vom Ausmaß der IM wurde häufig beschrieben [8, 9, 14, 25, 36–54]. In einem Fall einer „double depression“ konnte die EKT die depressiven Episoden lindern, nicht jedoch die Dysthymie [55].

Im Vergleich zu unipolar depressiven Störungen liegen zur Behandlung bipolarer Störungen etwas weniger Daten für den Einsatz von EKT bei Menschen mit IM vor. Die vorliegenden Publikationen berichten aber ebenfalls über erfolgreiche Anwendungen bei diesem Personenkreis [7, 9, 14, 19, 25–27, 36, 43, 49, 50, 56–67], sowohl bei depressiven als auch bei manischen oder gemischten Episoden.

### Schizophrenie, schizoaffektive Störung und andere psychotische Syndrome

Eine psychotische Symptomatik wird bei 4,8 % der Menschen mit IM diagnostiziert [34], eine deutlich höhere Anzahl (21 %) erhält Antipsychotika [35]. Auch wenn sich die EKT als Augmentationsstrategie bei einer Clozapin-resistenten Schizophrenie bei Menschen ohne IM wissenschaftlich etabliert hat [68, 69], liegen nur vereinzelte Fallberichte zur EKT bei Menschen mit IM und einer psychotischen Störung vor. Diese beschreiben – mit einer Ausnahme [6] – ein gutes Ansprechen auf die psychotische Symptomatik, teils über lange Zeiträume hinweg [8–11, 15, 26, 56, 57, 70–78, 134]. Dies gilt auch für schizoaffektive Störungen von Menschen mit IM [14, 36, 56, 57].

### Katatonie

Bei Menschen mit IM stellt die Katatonie eine immer häufigere EKT-Indikation dar, überwiegend als isolierte Störung [16, 20, 23, 29, 57, 79–97]. Katatone Syndrome wurden bei Menschen mit IM aber auch im Zusammenhang mit einer unipolaren Depression [42, 86], einer bipolaren Störung [19, 26, 57, 64], einer Psychose/Schizophrenie [9, 12, 15, 26, 28, 56, 70, 74, 78, 98] und einer schizoaffektiven Störung [14, 27] beschrieben und mit EKT erfolgreich behandelt. Auch Menschen mit Down-Syndrom erleiden häufiger katatone Störungen, teils im Rahmen eines akut oder subakut verlaufenden neuropsychiatrischen Syndroms, das als „down syndrome regression disorder (DSRD)“ beschrieben wird. Einige Autor:innen ([99]; Übersicht in [100, 101]) berichten hier in Fallserien von einer sehr guten Wirksamkeit der EKT.

Eine Sonderstellung nehmen katatone Syndrome bei ASS ein. Beide Störungen treten bei Menschen mit IM gehäuft auf und überlappen sich teilweise [102]. Unabhängig von dem Grad der IM kann eine EKT bei der Komorbidität ASS und Katatonie zu einer massiven Reduktion stereotyper, eigen- und fremdaggressiver Verhaltensweisen führen [13, 18, 21, 93, 94, 103–115].

### Therapieresistente schwere Verhaltensstörungen/selbstverletzendes Verhalten

Die Behandlung schwerer Verhaltensstörungen, im wesentlichen SVV, ist komplex. Zu den derzeit effektivsten Behandlungsmethoden gehören umfassende psychopharmakologische und verhaltenstherapeutische Ansätze [116]. Gerade die hochfrequenten und intensiven SVV bleiben jedoch häufig therapierefraktär. In dieser Indikation wurde die EKT in einzelnen Fällen erfolgreich angewendet [11, 23, 26, 29, 62, 64, 77, 85, 88–90, 96]. Fallberichte aus dem kinder- und jugendpsychiatrischen Bereich schildern teils extrem rasche Behandlungserfolge bei schwerstem SVV [86, 117, 135]. Inzwischen liegen Fallserien vor, die erfolgreiche Behandlungen über mehrere Jahre mit > 300 EKTs pro Patient:in schildern [24], unabhängig von der genauen Ätiologie der Verhaltensstörung.

### Nebenwirkungen

Die bei den typischen Patientengruppen ohne IM am häufigsten auftretenden Nebenwirkungen wie Kopf- und Muskelschmerzen, Übelkeit und temporäre kognitive Störungen [1] werden in den Berichten über EKT bei Menschen mit IM kaum erwähnt. Eine graduelle Verschlechterung der Kognition aufgrund der Grunderkrankung kann nicht immer sicher gegen eine EKT-induzierte kognitive Störung abgegrenzt werden [118]. Als Nebenwirkungen einer EKT wurden bei Menschen mit IM in Einzelfällen eine temporäre Einschränkung der Mobilität [119], ein überwachungspflichtiges, aber reversibles Pulmonalödem [120] und ein kurzzeitiges postinterventionelles Fieber ohne Infektionsnachweis beschrieben [61]. Möglicherweise kommt es im Vergleich zu Patient:innen ohne IM häufiger zu prolongierten Anfällen [8, 57, 87] und tardiven Anfällen [30, 121]. Als eine bisher unbeschriebene Nebenwirkung wurde eine Gewichtszunahme nach erfolgreicher EKT berichtet, weil die katatone Hyperaktivität reduziert wurde bei gleichbleibender Kalorienaufnahme [122].

## Diskussion

### Indikationen

In der Literatur finden sich vielfache Belege für den erfolgreichen Einsatz der EKT bei Menschen mit IM, wie in diesem Artikel dargelegt wird.

Fallberichte und Fallserien zur Anwendung der EKT bei IM zeigen ein breites Spektrum an möglichen Indikationen. Die häufigsten umfassen – unabhängig von der Ursache der IM – katatone Syndrome und ähnlich wie bei Menschen ohne IM schwere und/oder therapieresistente affektive, vor allem depressive, und psychotische Störungen. Die relative Häufigkeit der Indikation Katatonie, die in unserer Studie – teils in Kombination mit weiteren Diagnosen – die häufigste Indikation ausmacht, stellt im Vergleich zur EKT bei Menschen ohne IM eine Besonderheit dar. Während in Artikeln vor 2015 ähnliche EKT-Indikationen wie in Populationen ohne IM im Vordergrund standen, mehren sich in den letzten 10 Jahren die Berichte über Katatonie als Behandlungsindikation bei Menschen mit IM, vor allem wenn eine komorbide ASS vorliegt. So berichteten Collins et al. 2012 [123] in einer Übersicht von 72 Patient:innen mit IM, die mit EKT behandelt wurden, dass 58 % der Indikationen affektive Störungen ausmachten, gefolgt von der schizoaffektiven Störung (10 %), der Schizophrenie (8 %) und Verhaltensstörungen und der Katatonie mit einem Anteil von jeweils 6 %. Die Katatonie scheint in den letzten Jahren mit der Etablierung als eigenständige Entität losgelöst von der Schizophrenie in DSM‑5 und ICD-11 mehr ins Bewusstsein der Behandelnden gerückt zu sein. Darüber hinaus haben vor allem die Publikationen der Gruppe um Lee Wachtel [109] die Indikationen Katatonie und SVV bei – überwiegend sehr jungen – Menschen mit ASS und IM einem breiteren Fachpublikum bekannt gemacht. Bei vielen Patient:innen mit ASS und einer Katatonie wurden zusätzlich überlappende affektive [111] oder psychotische („iron triangle“ [72, 91]) Störungen wie auch Tic-Störungen diagnostiziert, was die vielfältigen klinischen Erscheinungsformen, aber auch die Schwierigkeiten der diagnostischen Einordnung widerspiegelt. In einer aktuellen Metaanalyse wurde aufgezeigt, dass von 15.434 Patient:innen mit ASS und Katatonie nur 22 mit einer EKT behandelt wurden [124]. Im klinischen Alltag bleiben katatone Symptome weiterhin häufig unterdiagnostiziert, und es kommt bei verspäteter Diagnose konsekutiv zu einer verspäteten antikatatonen Behandlung [125].

Eine der schwersten Formen stereotyper Verhaltensstörungen von Menschen mit IM ist SVV [126]. SVV ist bei der Doppeldiagnose ASS und IM mit ca. 38 % Betroffenen häufig [127] und umschließt eine große Gruppen an Verhaltensauffälligkeiten, wie Kopf-gegen-die-Wand-Schlagen, gegen sich selbst gerichtetes Schlagen, Stoßen, Kratzen, Reiben, Kneifen und Beißen [128]. In einer kleinen Subgruppe von Patient:innen tritt selbstverletzendes Verhalten in hoher Frequenz stakkatoartig auf und kann zu schweren Verletzungen z. B. des Augapfels, einer Netzhautablösung oder Hirnblutungen führen [128, 129]. Diese Verhaltensmuster müssen von selbstverletzendem Verhalten z. B. im Rahmen einer emotional-instabilen Persönlichkeitsstörung oder temporären Verhaltensauffälligkeiten bei sich neurotypisch entwickelnden Personen abgegrenzt werden. Die Ätiologie von SVV bei Menschen mit IM bleibt oft unklar. Teils wird SVV mit der IM bzw. der die IM bedingenden Erkrankung oder neuronalen Entwicklungsstörung in Verbindung gebracht, teils aber auch als klinischer Ausdruck einer komorbiden Katatonie gedeutet. So finden sich Fallbeschreibungen von verschiedenen Patient:innen mit sehr ähnlicher SVV-Symptomatik, die jeweils unter EKT sistierte und von manchen Autor:innen als Verhaltensstörung im Rahmen der IM (teils mit „funktionellem“ Charakter) und von anderen als Katatonie gedeutet werden. Zusammenfassend ähnelt EKT-responsives SVV am ehesten einer stereotypen Bewegungsstörung [96] – und ist wahrscheinlich häufig Ausdruck einer katatonen Störung.

### Alter

Das Durchschnittsalter der geschilderten Fälle ist mit knapp 28 Jahren im Vergleich zu Menschen ohne IM relativ gering. Während bei Patient:innen ohne IM aus verschiedenen Gründen – u. a. einer besseren Wirksamkeit im Vergleich zu Jüngeren – gerade ein Fokus auf der Behandlung eher älterer Menschen liegt und die EKT bei Kindern und Jugendlichen einer Ausnahme gleichkommt, ist dies aus den vorliegenden Daten für Menschen mit IM nicht abzuleiten. Bei den Behandlungen mit EKT aufgrund schwerster Verhaltensstörungen oder katatoner Syndrome handelte es sich vielmehr gerade um besonders junge, teils minderjährige Patient:innen.

### Wirksamkeit und Durchführung

Über alle Indikation hinweg findet sich eine sehr hohe Wirksamkeit der EKT, auch bei bis dahin therapieresistenten Störungen. Hier ist kein Unterschied zwischen Patient:innen mit IM im Vergleich zu Patient:innen ohne IM zu erkennen. Oft sind nur wenige Einzelbehandlungen bis zum Erreichen einer Remission erforderlich. Bei chronisch katatonen Zuständen werden anhaltende Remissionen aber auch erst nach einer Vielzahl an Behandlungen berichtet [22]. In vielen Fällen wird die EKT rückblickend als lebensrettende Maßnahme bewertet, nicht nur bei schweren depressiven Störungen mit Suizidalität und mangelnder Nahrungsaufnahme, sondern vor allem auch bei schwerem therapieresistentem SVV mit teils lebensbedrohlichen Verletzungen.

Die Rückfallrate nach Beenden der Indexserie ohne adäquate Rückfallprophylaxe ist wie bei Menschen ohne IM hoch [130]. In einigen Fällen, vor allem bei einer Katatonie, ist eine lange oder auch dauerhafte Erhaltungs-EKT mit hoher (wöchentlicher bis zweiwöchentlicher) Frequenz erforderlich [115].

### Limitationen

Die Datenlage ist mit den hier beschriebenen 208 Patientenberichten und fehlenden kontrollierten Studien naturgemäß limitiert. Zudem besteht die Gefahr eines Publikationsbias mit selektiven Schilderungen positiver Verläufe. Die Ergebnismessung basiert häufig auf multidisziplinären qualitativen Beurteilungen, da Selbstfragebögen nur eingeschränkt verwandt werden und klinische Skalen häufig nicht für Menschen mit IM genutzt werden können.

### Besondere Aspekte

In Zusammenschau der zur Verfügung stehenden Literatur entsteht der Eindruck, dass auch in Fällen mit klarer Indikation bei Menschen mit IM oft eine lange Phase von Therapieresistenz abgewartet wird, bevor die EKT angewendet wird. So erfolgt die Anwendung fast immer erst im Sinne einer Ultima Ratio, nachdem eine psychotrope Multimedikation und verschiedenste verhaltens- oder milieutherapeutische Interventionen erfolglos geblieben sind. Diese Problematik besteht in gewisser Weise auch in der Versorgung von Patient:innen ohne IM, scheint aber bei Menschen mit IM noch deutlich häufiger vorzukommen. Darüber hinaus finden sich weitere Hindernisse in Bezug auf den Zugang zur EKT bzw. Gründe für eine Zurückhaltung auf Seiten der Behandelnden [38]. Dies sind zum einen eine generelle diagnostische Unsicherheit bezogen auf psychische Störungen bei Menschen mit IM [45] und das in diesem Zusammenhang wahrscheinlich häufige Übersehen einer katatonen Symptomatik, für deren Behandlung im Vergleich zu anderen Störungsbildern neben der Benzodiazepin-Gabe kaum Alternativen zur EKT bestehen. Zum anderen besteht die Sorge, dass eine ohnehin bestehende kognitive Einschränkung unter einer EKT noch einmal zunehmen und die Patient:innen dauerhaft beeinträchtigen könne, was jedoch nicht der Datenlage entspricht [131]. Diese deutlich erschwerten Zugangswege zur EKT für Menschen mit IM entsprechen nicht dem Recht auf eine gleichwertige Behandlung gemäß der UN-Behindertenrechtskonvention Artikel 25 [132].

Während unter anderem in den USA und in den skandinavischen Ländern die EKT häufig ambulant angeboten wird, sodass die Patienten schon am Behandlungstag wieder ihre Alltagstätigkeiten aufnehmen können [86, 117], wird in Deutschland die EKT nahezu ausschließlich von psychiatrischen Kliniken im Rahmen stationärer Aufenthalte angeboten. Dies kann insbesondere die Behandlung von Menschen mit IM erschweren, da sie wiederholte Wechsel ihres Umfeldes und ihrer betreuenden Personen oft weniger gut tolerieren. Ambulante Behandlungsangebote für Menschen mit IM würden hier die Zugangswege vereinfachen [53].

## Schlussfolgerung

Die vorhandene Literatur spricht für ein sehr gutes Nutzen-Risiko-Verhältnis der EKT bei Menschen mit IM mit hohen Ansprechraten unabhängig von der Ursache der IM bzw. dem komorbiden Vorliegen einer ASS. Neben einer schweren affektiven oder psychotischen Symptomatik stellen vor allem katatone Syndrome eine Indikation für eine EKT bei Menschen mit IM dar. Die Ergebnisse unterstützen die aktuelle Stellungnahme der DGPPN zur EKT, in der dezidiert auch „katatone Syndrome“ und „therapieresistente schwere Verhaltensstörungen i. R. […] intellektueller Entwicklungsstörungen“ als EKT-Indikation aufgeführt werden [5]. Daher sollte die EKT bei der Behandlung dieser sonst schwer behandelbaren Erkrankungen bei Patienten mit IM häufiger und früher im Sinne einer „Optima Ratio“ in Betracht gezogen werden. Hier wären spezialisierte Zentren, welche auf die spezifischen Bedürfnisse von Menschen mit IM angepasst sind, sinnvoll.

## Supplementary Information

In der Supplement-eTabelle 1 sind die in dieser Studie ausgewerteten Kasuistiken und Fallserien im Einzelnen aufgeführt. Es finden sich dort personenbezogene Angaben zu Geschlecht, Alter, Schweregrad der Intelligenzminderung (IM) und Ursache der IM bzw. mit der IM assoziierten Erkrankungen. Bezogen auf die Elektrokonvulsionstherapie (EKT) sind dort die Indikation zur EKT, die Elektrodenposition, die EKT-Anzahl (differenziert nach der im jeweiligen Fallbericht beschriebenen ersten Serie, der EKT-Gesamtanzahl im Behandlungsverlauf inklusive eventueller Erhaltungs-EKT) und das Behandlungsergebnis (gebessert/unverändert/verschlechtert) aufgeführt. Unter „Ergebnisinstrument“ findet sich die Information, ob das Behandlungsergebnis rein klinisch oder mit Hilfe eines Tools erhoben wurde.

## Abkürzungen

ABC: Aberrant Behaviour Checklist
ABS: Adaptive Behaviour Scale
BDI: Beck-Depression-Inventar
BFCRS: Bush-Francis Catatonia Rating Scale
BPRS: Brief Psychiatric Rating Scale
CDRS‑R: Children’s Depression Rating Scale – revised
CGI: Clinical Global Impression Scale
C‑SHARP: Children’s Scale for Hostility and Aggression
CTONI: Comprehensive Test of Nonverbal Intelligence
CYBOCS: Childhood Yale-Brown Obsessive Compulsive Scale
EKT: Elektrokonvulsionstherapie
GAF: Global Assessment of Functioning
HAM‑D: Hamilton Depression Rating Scale
IED: Intermittent explosive disorder
IM: Intelligenzminderung
IQ: Intelligenzquotient
k. A.: Keine Angabe
MMSE: Mini Mental Status Examination
MNS: Malignes neuroleptisches Syndrom
RUL: Rechts unilateral
SVV: Selbstverletzendes Verhalten
YAPA-SIBS: Yale-Paris Self-injurious Behavior Scale
