# Supplementary material for: Electroconvulsive therapy in people with intellectual disability
Source: Nervenarzt. 2024 Aug 21;96(2):166–75. [Article in German] doi: 10.1007/s00115-024-01713-6 (PMC11876283; doi:10.1007/s00115-024-01713-6)
Supplement: Supplementary file 1 — In der Supplement-eTabelle 1 sind die in dieser Studie ausgewerteten Kasuistiken und Fallserien im Einzelnen aufgeführt. Es finden sich dort personenbezogene Angaben zu Geschlecht, Alter, Schweregrad der Intelligenzminderung (IM) und Ursache der IM bzw. mit der IM assoziierten Erkrankungen. Bezogen auf die Elektrokonvulsionstherapie (EKT) sind dort die Indikation zur EKT, die Elektrodenposition, die EKT-Anzahl (differenziert nach der im jeweiligen Fallbericht beschriebenen ersten Serie, der EKT-Gesamtanzahl im Behandlungsverlauf inklusive eventueller Erhaltungs-EKT) und das Behandlungsergebnis (gebessert/unverändert/verschlechtert) aufgeführt. Unter „Ergebnisinstrument“ findet sich die Information, ob das Behandlungsergebnis rein klinisch oder mit Hilfe eines Tools erhoben wurde. [file 115_2024_1713_MOESM1_ESM.docx]

| **Studie** | **Geschlecht (M=männlich; F=weiblich)** | **Alter** | **Schweregrad der IM** | **Ursache der IM/mit der IM assoziierte Erkrankung** | **EKT-Indikation** | **Elektroden-position** | **EKT-Anz.**  **1. Serie** | **EKT-Anz. gesamt (E=Erhaltungs-EKT)** | **Ergebnis** | **Ergebnis-instrument** |
| --- | --- | --- | --- | --- | --- | --- | --- | --- | --- | --- |
| Payne, 1968[9] | M | 38 | borderline (IQ=83) | k.A. | Bipolare Störung | k.A. | k.A. | k.A. | gebessert | k.A. |
|  | M | 31 | mild-moderate  (IQ= 50) | k.A. | Depression | k.A. | k.A. | k.A. | gebessert | k.A. |
|  | M | 19 | borderline  (IQ=75) | k.A. | Psychose, Katatonie | k.A. | k.A. | k.A. | unverändert | k.A. |
| Bates & Smeltzer, 1982[77] | M | 25 | severe  (IQ=21-25) | k.A. | Psychose, SVV | bilateral | 12 | 12 | gebessert | klinisch |
| Hermle & Oepen, 1986[28] | F | 19 | k.A. | k.A. | Katatonie (DD MNS) | bilateral | 12 | 12 | gebessert | klinisch |
| Knitter, 1986[6] | k.A.  (N = 5) | k.A. | k.A. („Oligo-phrenie“) | k.A. | Psychose („Propf-psychosen“) | k.A. | k.A. | k.A. | n=4 unverändert; n=1 verschlechtert | k.A. |
| Guze et al., 1987[60] | M | 21 | mild (IQ=65) | k.A. | Bipolare Störung (gemischt) | RUL | 8 | 8 | gebessert | (BDI initial) |
| Kearns, 1987[54] | M | 67 | moderate (IQ=35) |  | Depression | k.A. | 6 | 6 | gebessert | klinisch |
| Goldstein & Jensvold, 1989[52] | M | 68 | mild (IQ=63) | k.A. | Depression | RUL | 10 | 10 | gebessert | k.A. |
| Slack & Stoudemire, 1989[7] | M | 18 | mild | k.A. | Bipolare Störung, MNS | k.A. | 4 | k.A. | verschlechtert | k.A. |
| Warren et al., 1989[44] | F | 17 | k.A. | Down-Syndrom | Depression | k.A. | 14 | 14 | gebessert | klinisch |
|  | M | 24 | k.A. | Down-Syndrom | Depression | k.A. | 10 | 10 | gebessert | klinisch |
|  | M | 24 | k.A. | Down-Syndrom | Depression | k.A. | 5 | 5 | gebessert | klinisch |
| Day 1990[46] | M | 37 | moderate | k.A. | Depression | k.A. | k.A. | k.A. | gebessert | k.A. |
|  | F | 47 | borderline | k.A. | Depression, Schizophrenie | k.A. | k.A. | k.A. | gebessert | k.A. |
| Lazarus et al. 1990[47] | F | 32 | mild | Down-Syndrom | Depression | k.A. | 6 + E | k.A. | gebessert | k.A. |
|  | F | 50 | mild | Down-Syndrom | Depression | k.A. | 6 + E | k.A. | gebessert | k.A. |
| Merrill, 1990[40] | F | k.A. | profound | k.A. | Depression | k.A. | 10 | 10 | gebessert | klinisch |
| Karvounis et al., 1992[53] | M | 69 | moderate (IQ=40) | k.A. | Depression | bilateral | 11 | 11 | gebessert | klinisch |
| Puri et al., 1992[41] | M | 32 | mild (IQ=70) | k.A. | Depression | bilateral | k.A. + E | k.A. | gebessert | ABS |
| Jyoti Rao et al., 1993[15] | M | 18 | moderate | k.A. | Psychose, Katatonie | k.A. | 9 | 9 | unverändert | k.A. |
| Everman & Stoudemire, 1994[59] | M | 38 | mild | Klinefelter-Syndrom | Bipolare Störung (Manie) | RUL | 8 | 8 | gebessert | klinisch |
| Jancar & Gunaratne, 1994[55] | M | 28 | moderate (IQ=46) | k.A. | Depression (double depression) | k.A. | k.A. | k.A. | gebessert | klinisch |
|  | F | 38 | mild (IQ=55) | k.A. | Depression (double depression) | k.A. | k.A. | k.A. | gebessert | klinisch |
| Snowdon et al., 1994[63] | F | 69 | mild | k.A. | Bipolare Störung (Depression), Verhaltensauff. Schreien) | RUL, bilateral | 10 | 26 | gebessert | klinisch |
| Bebchuk et al., 1996[65] | M | 44 | profound | k.A. | k.A. („Demenz, Depression, Bipolare Störung“) | RUL | 6 + E | k.A. | gebessert | klinisch |
| Cutajar et al., 1998[50] | F | 23 | mild | k.A. | Depression | k.A. | 6 | 6 | gebessert | k.A. |
| Gabriel 1998[73] | F | 65 | moderate | k.A. | Psychose | k.A. | 15 + E | k.A. | gebessert | BPRS, GAF |
| Brasić et al., 1999[16] | M | 19 | k.A. | k.A. | Katatonie | k.A. | 25 | 25 | unverändert | klinisch |
| Chanpattana, 1999[75] | F | 31 | severe (IQ=30-35) | k.A. | Schizophrenie | bilateral | 6 + E | k.A. | gebessert | klinisch |
|  | M | 21 | moderate (IQ=52) | k.A. | Schizophrenie | bilateral | 21 + E | k.A. | gebessert | Klinisch (+BPRS, MMSE) |
|  | M | 45 | moderate | k.A. | Schizophrenie | bilateral | 4 + E | k.A. | gebessert | Klinisch (BPRS, MMSE) |
| Cutajar & Wilson, 1999[49] | M | k.A. | mild | k.A. | Depression | k.A. | 6 | 6 | gebessert | klinisch |
|  | F | k.A. | moderate | k.A. | Bipolare Störung (Depression) | k.A. | 6 | 6 | gebessert | klinisch |
|  | M | k.A. | moderate | k.A. | Bipolare Störung (Depression) | k.A. | 10 | 10 | gebessert | klinisch |
|  | F | k.A. | mild | k.A. | Depression | k.A. | 12 | 12 | gebessert | klinisch |
| Fink 1999[67] | F | 23 | mild-moderate | k.A. | Bipolare Störung (gemischt), Katatonie | k.A. | 3 | 3 | gebessert | k.A. |
| Fink 1999[134]133 | M | 14 | moderate | k.A. | Verhaltenauff. (SVV, Schreien) | k.A. | 16 | 16 | gebessert | k.A. |
| Gothelf et al., 1999[10] | F | 35 | mild | Velo-kardio-faziales Syndrom | Schizophrenie | k.A. | k.A. | k.A. | unverändert | k.A. |
|  | F | 30 | mild | Velo-kardio-faziales Syndrom | Schizophrenie | k.A. | k.A. | k.A. | unverändert | k.A. |
| Ruedrich & Alamir 1999[48] | F | 44 | mild | k.A. | Depression | k.A. | 5 | 5 | gebessert | k.A. |
| Zaw et al., 1999[57] | M | 56 | mild | k.A. | Schizoaffektive Störung | bilateral | 9 | 9 | gebessert | klinisch |
|  | M | 18 | Moderate (IQ=40) | k.A. | Bipolare Störung | bilateral | 13 + E | 17 | gebessert | klinisch |
|  | M | 29 | mild | k.A. | Katatonie | bilateral | 17 + E | 45 | gebessert | klinisch |
|  | M | 64 | mild | k.A. | Schizoaffektive Störung | bilateral | k.A. | 28 | gebessert | klinisch |
|  | F | 25 | moderate | k.A. | Bipolare Störung, Katatonie | bilateral | 3 + E | 31 | gebessert | klinisch |
| Zaw et al., 1999[121] | M | 14 | borderline (moderate learning difficulty) | Autismus | Katatonie | bilateral | 13 | 13 | gebessert | klinisch |
| Van Waarde et al., 2000[43] | F | 67 | mild | k.A. | Bipolare Störung (Depression) | bilateral | 9 | 21 | gebessert | klinisch |
|  | M | 55 | moderate (IQ=45) | k.A. | Depression | k.A. | 9 | 9 | gebessert | klinisch |
| Aziz et al., 2001[27] | M | 22 | moderate | k.A. | Bipolare Störung, MNS | bilateral | 6 | 6 | gebessert | klinisch |
|  | F | 39 | moderate | k.A. | Schizoaffektive Störung, Katatonie | bilateral | 11 | 11 | gebessert | klinisch |
| Chopra & Sinha, 2002[25] | M | 19 | mild (IQ=58) | k.A. | Manie | bilateral | 6 | 6 | gebessert | klinisch |
|  | M | 23 | moderate (IQ=42) | k.A. | Psychose | bilateral | 8 + E | k.A. | gebessert | klinisch |
|  | M | 26 | mild (IQ=62) | k.A. | Bipolare Störung (Manie) | bilateral | 8 | 8 | gebessert | klinisch |
|  | M | 18 | mild (IQ=68) | k.A. | Manie | bilateral | 7 | 7 | gebessert | klinisch |
|  | M | 30 | mild (IQ=54) | k.A. | Depression | bilateral | 10 + E | k.A. | gebessert | klinisch |
| Friedlander & Solomons, 2002[14] | F | 41 | mild | k.A. | Schizoaffektive Störung | bilateral | 11 + E | k.A. | gebessert | CGI |
|  | F | 38 | mild | k.A. | Depression | bilateral | 3 | 3 | gebessert | CGI |
|  | M | 36 | mild | k.A. | Schizoaffektive Störung, MNS | bilateral | 3 | 3 | unverändert | CGI |
|  | F | 46 | moderate | k.A. | Org. aff. Störung | bilateral | 4 | 4 | gebessert | CGI |
|  | M | 35 | severe | k.A. | Depression | bilateral | 6 + E | k.A. | gebessert | CGI |
|  | M | 29 | mild | k.A. | Bipolare Störung (gemischt) | bilateral | 8 | 8 | gebessert | CGI |
|  | M | 47 | mild | Turner-Syndrom | Schizoaffektive Störung, Katatonie | bilateral | 9 | 9 | unverändert | CGI |
|  | M | 21 | mild | k.A. | Bipolare Störung (Manie) | bilateral | 10 | 10 | gebessert | CGI |
|  | M | 17 | mild | k.A. | Schizoaffektive Störung | bilateral | 15 | 15 | unverändert | CGI |
|  | M | 37 | moderate | k.A. | Bipolare Störung (gemischt) | bilateral | 4 | 4 | gebessert | CGI |
| Gensheimer et al., 2002[51] | M | 15 | k.A. | Down-Syndrom | Depression | bilateral | 4 | 4 | gebessert | CDRS-R, CGI |
| Little et al., 2002[38] | F | 30 | k.A. | k.A. | Depression | bilateral | 12 | 12 | gebessert | k.A. |
|  | F | 52 | mild | k.A. | Depression | bilateral | 7 | 7 | gebessert | k.A. |
|  | M 50 | 50 | k.A. | k.A. | Depression | bilateral | 6 | 6 | gebessert | k.A. |
| Kessler, 2004[36] | M | 22 | mild | k.A. | Depression | k.A. | 9 | 9 | gebessert | klinisch |
|  | F | 28 | mild | k.A. | Bipolare Störung (Rapid cycling) | k.A. | 6 | 6 | gebessert | klinisch |
|  | F | 36 | mild | k.A. | Schizoaffektive Störung | k.A. | 7 | 7 | gebessert | klinisch |
|  | F | 35 | mild | k.A. | Bipolare Störung (Manie) | k.A. | 4 | 4 | gebessert | klinisch |
| Reinblatt et al., 2004[8] | N = 20  (11 M, 9 F) | k.A. | 7 mild (50-70)  5 moderate (35-50)  1 severe (20-35)  7 profound (<20) | k.A. | 12 affektive Störungen  6 psychotische Störungen  2 „intermittend explosive disorder“ (IED) | bilateral, bifrontal | k.A. | k.A. | gebessert  (aff + psych)  unverändert (IED) | ABC, CGI-S |
| Ghaziuddin et al., 2005[114] | M | 17 | mild | Autismus | Katatonie | bilateral | 18 | 18 | gebessert | k.A. |
| Tan et al., 2006[104] | M | 20 | k.A. | Autismus | Katatonie | RUL | 17 | 17 | gebessert | BFCRS |
| Bailine & Petraviciute, 2007[106] | M | 19 | mild | Autismus | Katatonie | bifrontal | 19 | 19 | gebessert | klinisch |
|  | M | 19 | mild | Autismus | Katatonie | bifrontal | 19 | 19 | gebessert | klinisch |
| Mackay & Wilson, 2007[39] | F | k.A. | mild | k.A. | Depression | k.A. | k.A. | k.A. | gebessert | k.A. |
| Myers & Dinwiddie, 2007[11] | M | 20 | mild | FG-Syndrom | Schizophrenie | bilateral | k.A. + E | k.A. | gebessert | klinisch |
| Kakooza-Mwesige et al., 2008[105] | M | 20 | mild (IQ=86) | Autismus | Katatonie | k.A. | 20 + E über 6 Jahre | k.A. | gebessert | klinisch |
| Wachtel et al., 2008[23] | F | 16 | k.A. | Autismus | Katatonie, Verhaltensauff. (SVV) | RUL, bilateral | 12 + E | k.A. | gebessert | klinisch |
| Ligas et al., 2009[19] | F | 39 | borderline | k.A. | Bipolare Störung, Katatonie | bifrontal | 8 + E | k.A. | gebessert | klinisch |
| Tripp & Jacobson, 2009[135]134 | M | 24 | borderline | FG-Syndrom | k.A. („mood instabilty“) | k.A. | k.A. | 180 | gebessert | klinisch |
| Wachtel et al., 2009[136] 135 | M | 8 | k.A. | Autismus | Verhaltensauff. (SVV) | bitemporal | 15 | 15 | gebessert | klinisch |
| Ghaziuddin et al., 2010[94] | M | 18 | mild | Autismus | Katatonie | bilateral | 12 | 12 | gebessert | klinisch |
|  | M | 16 | moderate | Autismus | Katatonie | bilateral | 29 + E | k.A. | gebessert | klinisch |
| Wachtel et al., 2012; Wachtel, Crawford, Dhossche, et al., 2010[86], [132] 131 | M | 15 | borderline- mild | (cerebellar dysgenesis) | Katatonie | bilateral | 6 + E | 67 | gebessert | klinisch  CTONI |
| Wachtel, Griffin, & Reti, 2010[119] | M | 19 | mild | Autismus | Depression, Katatonie, Verhaltensauff. (SVV) | bitemporal | 7 + E | k.A. | gebessert | klinisch |
| Wachtel et al., 2010[111] | M | 14 | mild | Autismus | Katatonie | RUL, dann bitemporal | k.A. (+ E) | k.A. | gebessert | klinisch |
| Jap & Ghaziuddin, 2011[97] | F | 16 | borderline | Down-Syndrom | Katatonie | bitemporal | 15 + E | k.A. | gebessert | klinisch |
| Palm et al., 2011[70] | M | 35 | k.A. | (Corpus callosum-Aplasie) | Schizophrenie, Katatonie | rechts-occipital | 8 | 8 | gebessert | klinisch |
| Wachtel et al., 2011[117] | M | 11 | mild-moderate | Autismus | Bipolare Störung, SVV | bitemporal | 8 + E | k.A. | gebessert | CYBOCS, ABC |
| Chathanchirayil & Bhat, 2012[58] | F | 37 | moderate | k.A. | Bipolare Störung (Rapid cycling) | RUL/ bilateral | k.A. + E | k.A. | gebessert | klinisch |
| Consoli et al., 2012[26] | M | 14 | k.A. | ProDH-Mutation(k.A.) | Schizophrenie, Katatonie, Verhaltensauff. (SVV/Aggression) | bilateral | 12 | 12 | gebessert | SVV/Aggression-Score („Lambrey method“) |
|  | F | 12 | k.A. | k.A. | Bipolare Störung (gemischt), Katatonie, Verhaltensauff. (SVV/Aggression) | bilateral | 16 + E | k.A. | gebessert | SVV/Aggression-Score („Lambrey method“) |
|  | F | 14 | k.A. | Deletion 13q34 | Bipolare Störung (Depression), Verhaltensauff. (SVV/Aggression) | bilateral | 22 | 22 | gebessert | SVV/Aggression-Score („Lambrey method“) |
|  | M | 13 | k.A. | k.A. | Manie, Verhaltensauff. (SVV/Aggression) | bilateral | 26 | 26 | gebessert | SVV/Aggression-Score („Lambrey method“) |
| Shah et al., 2012[31] | M | 18 | mild | Tuberkulöse Meningitis | Sonstige (Epilepsie – breakthrough seizures) | k.A. | k.A. + E | 52 | gebessert | klinisch  (Anzahl d. Anfälle) |
| Siegel et al., 2012[62] | M | 16 | moderate | Autismus | Bipolare Störung (gemischt), Verhaltensauff. (SVV) | bilateral | 10 | 10 | gebessert | klinisch |
| Majeske et al., 2013[61] | M | 27 | mild -moderate | k.A. | Bipolare Störung (Manie) | bilateral | 7 | 7 | gebessert | klinisch |
| Wachtel & Shorter, 2013[98] | F | 13 | k.A. | Autismus | Psychose, Katatonie („Iron Triangle“) | bilateral | 12 | 12 | gebessert | BFCRS, klinisch |
| Dhossche, 2014[18] | M | 10 | Borderline (IQ 80) | Autismus | Katatonie | bilateral | 14 | 14 | gebessert | klinisch |
| Haq & Ghaziuddin, 2014[96]  (2. Pat. Anderweitig publiziert) | F | 15 | moderate | Autismus | Katatonie | bilateral | 17 + E | k.A. | gebessert | klinisch |
| Torr & D’Abrera, 2014[42] | F | 23 | mild | Down-Syndrom | Depression, Katatonie | bilateral | 5 + E | k.A. | gebessert | klinisch |
| Vowels et al., 2014[71] | F | 18 | moderate | k.A. | Schizophrenie | bitemporal | 8 + E | k.A. | gebessert | klinisch |
| Wachtel et al., 2014[130] | M | 12 | k.A. | Autismus | Katatonie (SVV) | bitemporal | k.A. | 53 | gebessert | klinisch |
| Dare & Rasmussen, 2015[56] | F | 62 | k.A. | k.A. | Bipolare Störung, Verhaltensauff. (recurrent behavioral dyscontrol) | bitemporal | k.A. + E | 205 | gebessert | k.A. |
|  | F | 65 | mild | k.A. | Schizophrenie, Katatonie | bitemporal | k.A. + E | 207 | gebessert | k.A. |
|  | M | 71 | moderate | k.A. | Demenz, Verhaltensauff. („recurrent behavioral dyscontrol“) | bitemporal/  RUL | k.A. + E | 187 | gebessert | k.A. |
|  | F | 29 | mild | k.A. | Schizoaffektive Störung, Verhaltensauff. („recurrent behavioral dyscontrol“) | bitemporal | k.A. + E | 181 | gebessert | k.A. |
| Ghaziuddin et al., 2015[95] | F | 15 | mild | Down-Syndrom | Katatonie | bilateral | 26 + E | k.A. | gebessert | BFCRS |
|  | F | 16 | moderate | Down-Syndrom | Katatonie | bilateral | k.A. + E | 86 | gebessert | BFCRS |
|  | M | 16 | moderate | Down-Syndrom | Katatonie | bilateral | k.A. + E | 76 | gebessert | BFCRS |
|  | M | 18 | moderate | Down-Syndrom | Katatonie | bilateral | 12 | 12 | gebessert | BFCRS |
| Poser & Trutia, 2015[81] | F | 25 | mild | Prader-Willi-Syndrom | Katatonie | bifrontal, bitemporal | 8 | 8 | gebessert | klinisch |
| Wachtel et al., 2015[64] | M | 18 | mild | Autismus | Bipolare Störung, Katatonie | bitemporal | 34 + E | k.A. | gebessert | klinisch |
| Winarni et al.,  2015[118] | M | 20 | mild (IQ=57) | Fragiles X-Premutation | Katatonie | bilateral | 21 + E | k.A. | gebessert | k.A. |
| Baldinger-Melich et al., 2016[12] | M | 42 | k.A. | k.A. | Katatonie, Schizophrenie | bilateral | 15 | 15 | unverändert | BFCRS |
| Mircher et al., 2017[99] | F | k.A. | k.A. | Down-Syndrom | Katatonie („acute regression“) | k.A. | 6 | 6 | gebessert | klinisch |
| Sajith et al., 2017[90] | M | 21 | mild | Autismus | Katatonie, Verhaltensauff. (SVV) | RUL | 11 + E | k.A. | gebessert | ABC |
|  | M | 23 | moderate | Autismus | Katatonie, Verhaltensauff. (SVV) | RUL | 12 + E | k.A. | gebessert | ABC |
| Desarkar et al., 2018[78] | F | 26 | moderate | k.A. | Schizophrenie, Katatonie | bilateral | 10 + E | k.A. | gebessert | BPRS + BFCRS |
| Lingeswaran & Gopal, 2018[37] | F | 40 | k.A. | Papillon-Lefèvre-Syndrom | Depression | bilateral | 10 | 10 | gebessert | HAM-D |
| Withane & Dhossche, 2018[110] | M | 16 | severe | Autismus | Katatonie | k.A. | 2 + E | k.A. | gebessert | klinisch |
|  | M | 13 | moderate | Autismus | Katatonie | bilateral | 12 | 12 | gebessert | klinisch |
| Wachtel 2018[109] | M | 15 | mild | Autismus | Katatonie | k.A. | k.A. | k.A. | gebessert | k.A. |
|  | M | 14 | mild | Autismus | Katatonie (maligne) | RUL | k.A. + E | k.A. | gebessert | k.A. |
|  | F | 17 | k.A. | Autismus | Katatonie (SVV) | bilateral | k.A. + E | k.A. | gebessert | k.A. |
| Cardinale et al., 2019[74] | F | 23 | k.A. | Down-Syndrom | Psychose, Katatonie („Down Syndrome disintegrative disorder“) | k.A. | k.A. | k.A. | gebessert | k.A. |
| González-Romero et al., 2019[29] | M | 9 | moderate | Autismus | Verhaltensauff. (SVV), MNS | bitemporal | 12 + E | k.A. | gebessert | CGI |
| Machado et al., 2019[88] | M | 17 | k.A. | Autismus | Katatonie, Verhaltensauff. (SVV) | k.A. | 4 | 4 | gebessert | klinisch |
| Miles et al., 2019[79] | M | 18 | moderate | Down-Syndrom | Katatonie | bifrontal | k.A. | k.A. | gebessert | BFCRS |
|  | F | 19 | moderate – severe | Down-Syndrom | Katatonie | k.A. | k.A. + E | k.A. | gebessert | BFCRS |
|  | F | 33 | keine | Down-Syndrom | Katatonie | k.A. | 17 + E | k.A. | gebessert | BFCRS |
|  | F | 25 | keine | Down-Syndrom | Katatonie | k.A. | 19 | 19 | gebessert | BFCRS |
|  | F | 19 | keine | Down-Syndrom | Katatonie | k.A. | k.A. + E | k.A. | gebessert | BFCRS |
|  | F | 26 | keine | Down-Syndrom | Katatonie | k.A. | k.A. | k.A. | gebessert | BFCRS |
| Stumpf et al., 2019[30] | F | 42 | k.A. | k.A. | Delir | bilateral | 10 | 10 | gebessert | klinisch |
| Bird et al., 2020, 2023[107], [108] | M | 30 |  | Autismus, SIB, Depression | Katatonie | Bilateral | k.A. | >100 | gebessert | Anzahl Aggressiven Verhaltens u. Rating durch Betreuer |
| Naguy et al., 2020[89] | M | 10 | moderate | Autismus | Verhaltensauff. (SVV, Fremdaggression) | bitemporal | 12 | 12 | gebessert | ABC, YAPA-SIBS, C-SHARP |
| Suman et al., 2020[82] | F | 20 | k.A. | k.A. | Katatonie | bitemporal | 7 | 7 | gebessert | BFCRS |
| Triplett et al., 2020[83] | F | 33 | mild | Autismus | Katatonie | bilateral | 9 | 9 | gebessert | Klinisch (BFCRS) |
| Park et al., 2020[126] | F | 17 | k.A. | Autismus | Katatonie | bilateral | 12 | 24 | gebessert | klinisch |
| Burns et al., 2021[93] | M | 18 | keine | Autismus | Katatonie | k.A. | 8 + E | k.A. | gebessert | klinisch |
|  | M | 17 | profound | Autismus | Katatonie | bilateral | 8 + E | k.A. | gebessert | klinisch |
| Mehra & Padhy, 2021[92] | M | 17 | k.A. | k.A. | Katatonie | bilateral | 14 | 14 | gebessert | BFCRS |
| Mormando et al., 2021[80] | M | 21 | k.A. | Autismus | Katatonie | bitemporal | k.A. + E | k.A. | gebessert | klinisch |
| Mormando et al., 2021b[103] | M | 19 | k.A. | Autismus | Katatonie | RUL, bitemporal | k.A. + E | 45 | gebessert | BFCRS |
| Vieira et al., 2021[84] | F | 23 | severe | Pitt-Hopkins-Syndrom, Autismus | Katatonie | bilateral | 13 | 13 | gebessert | BFCRS |
| Ghaziuddin et al., 2021[115] | N=9 (F=5, M=4) | 16-20 | k.A. |  | Katatonie (in Erhaltungs-EKT, pausiert durch Covid-19 mit klinischer Verschlechterung, dann Wiederaufnahme von E-EKT) | bilateral |  |  | gebessert |  |
| Wachtel, 2021[24], 2. Case bereits publiziert | M | 27 | k.A. | Autismus | Katatonie | k.A. | k.A. + E | >100 | gebessert | k.A. |
| Anthonio et al., 2022[22] | M | 22 | k.A. | Down-Syndrom | Depression, Katatonie | k.A. | k.A. + E | >100 | gebessert | klinisch/  BFCRS |
| Smith et al., 2022[13] | M | 14 | k.A. | Autismus | Katatonie, SVV | bitemporal | 14 | 14 | gebessert | CGI |
|  | M | 20 | k.A. | Autismus | Katatonie, SVV | bitemporal | 21 | 21 | gebessert | CGI |
|  | F | 33 | k.A. | Autismus | Katatonie, Psychose | bitemporal | 8 | 15 | gebessert | CGI |
|  | M | 20 | k.A. | Autismus | SVV | bitemporal | 10 + E | k.A. | gebessert | CGI |
|  | M | 26 | k.A. | Autismus | Katatonie | bitemporal | 8 + E | k.A. | gebessert | CGI |
|  | M | 17 | k.A. | Autismus | Katatonie, SVV | bitemporal | 5 + E | k.A. | gebessert | CGI |
|  | M | 21 | k.A. | Autismus | Katatonie, SVV | bitemporal | 6 | k.A. | gebessert | CGI |
|  | M | 16 | k.A. | Autismus | Katatonie | bitemporal | 14 + E | k.A. | gebessert | CGI |
|  | M | 22 | k.A. | Autismus | Katatonie | bitemporal | 14 | 14 | gebessert | CGI |
|  | M | 29 | k.A. | Autismus | Katatonie, SVV | bitemporal | E | k.A. | gebessert | CGI |
|  | M | 16 | k.A. | Autismus | Katatonie | bitemporal | E | k.A. | gebessert | CGI |
|  | M | 29 | k.A. | Autismus | Katatonie, Psychose | bitemporal | 8 | k.A. | unverändert | CGI |
|  | F | 42 | k.A. | k.A. | Katatonie | bitemporal | 12 + E | k.A. | gebessert | CGI |
|  | F | 13 | k.A. | k.A. | Schizoaffektive Störung | bitemporal | 18 + E | k.A. | gebessert | CGI |
|  | M | 18 | k.A. | k.A. | Katatonie | bitemporal | 6 | 6 | gebessert | CGI |
|  | F | 55 | k.A. | k.A. | Katatonie, Depression | RUL | 15 | 35 | gebessert | CGI |
|  | F | 37 | k.A. | k.A. | Katatonie | bitemporal | 14 | 14 | unverändert | CGI |
|  | F | 16 | k.A. | k.A. | Psychose | bifrontal | 10 + E | k.A. | gebessert | CGI |
|  | M | 36 | k.A. | k.A. | Katatonie, Psychose | bitemporal | 15 + E | k.A. | gebessert | CGI |
|  | F | 30 | k.A. | k.A. | Katatonie | bitemporal | 8 + E | k.A. | gebessert | CGI |
|  | F | 26 | k.A. | k.A. | Psychose, Depression | bitemporal | 10 + E | k.A. | gebessert | CGI |
|  | F | 17 | k.A. | k.A. | Psychose, Depression | bitemporal | 9 | 9 | gebessert | CGI |
|  | F | 64 | k.A. | k.A. | Bipolare Störung (Depression) | RUL | 5 + E | k.A. | gebessert | CGI |
|  | F | 32 | k.A. | k.A. | Katatonie | RUL | 6 | 6 | gebessert | CGI |
|  | F | 24 | k.A. | k.A. | Katatonie | bitemporal | 15 | 15 | gebessert | CGI |
| Free et al., 2023[123]122 | F | 36 | k.A. | Autismus | Katatonie | bitemporal | 15 | 15 | gebessert | BFCRS |
| Purgianto & Nickl-Jokschat, 2023[122]121 | F | 40 | moderate | k.A. | Katatonie | RUL | 2 | 2 | abgebrochen wg. tardiven Anfalls | - |
|  | F | 63 | k.A. | k.A. | Katatonie | bitemporal | 4 | 4 | abgebrochen wg. tardiven Anfalls | - |

eTabelle 1

Abkürzungsverzeichnis: ABC: Aberrant Behaviour Checklist; ABS: Adaptive Behaviour Scale; BDI: Beck-Depression- Inventar; BFCRS: Bush-Francis Catatonia Rating Scale; BPRS: Brief Psychiatric Rating Scale; CDRS-R: Children´s Depression Rating Scale – revised; CTONI: Comprehensive Test of Nonverbal Intelligence; CYBOCS: Childhood Yale-Brown Obsessive Compulsive Scale; CGI: Clinical Global Impression Scale; C-SHARP: Children´s Scale for Hostility and Aggression; EKT: Elektrokonvulsionstherapie; GAF: Global Assement of Functioning; HAM-D: Hamilton Depression Rating Scale; IED: intermittend explosive disorder; IM: Intelligenzminderung; IQ: Intelligenzquotient; k.A.: keine Angabe; MMSE: Mini Mental Status Examination; MNS: malignes neuroleptisches Syndrom; RUL: rechts unilateral; SVV: selbstverletzendes Verhalten; YAPA-SIBS: Yale-Paris Self-injurious Behavior Scale
